# Growth in Turface® clay permits root hair phenotyping along the entire crown root in cereal crops and demonstrates that root hair growth can extend well beyond the root hair zone

**DOI:** 10.1186/s13104-015-1108-x

**Published:** 2015-04-12

**Authors:** Travis L Goron, Sophia Watts, Charles Shearer, Manish N Raizada

**Affiliations:** Department of Plant Agriculture, University of Guelph, Guelph, ON N1G 2W1 Canada

**Keywords:** Root hair, Wheat, Maize, Finger millet, Cereal, Root hair zone, Turface®, Clay, Crown root

## Abstract

In cereal crops, root hairs are reported to function within the root hair zone to carry out important roles in nutrient and water absorption. Nevertheless, these single cells remain understudied due to the practical challenges of phenotyping these delicate structures in large cereal crops growing on soil or other growth systems. Here we present an alternative growth system for examining the root hairs of cereal crops: the use of coarse Turface® clay alongside fertigation. This system allowed for root hairs to be easily visualized along the entire lengths of crown roots in three different cereal crops (maize, wheat, and finger millet). Surprisingly, we observed that the root hairs in these crops continued to grow beyond the canonical root hair zone, with the most root hair growth occurring on older crown root segments. We suggest that the Turface® fertigation system may permit a better understanding of the changing dynamics of root hairs as they age in large plants, and may facilitate new avenues for crop improvement below ground. However, the relevance of this system to field conditions must be further evaluated in other crops.

## Discussion

### Do cereal crops have a canonical root hair zone?

**Figure 1:**
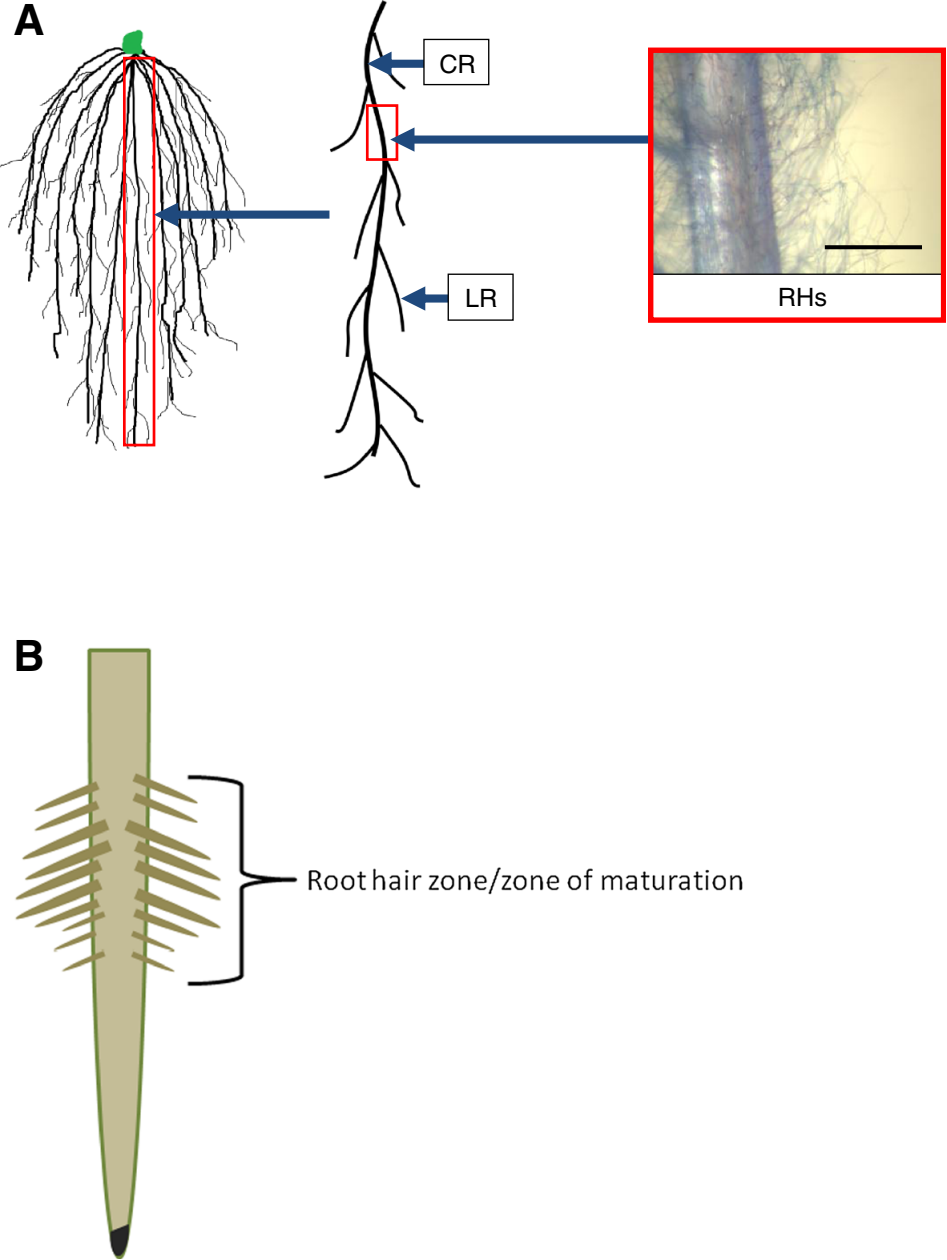
Root hairs as they appear on the primary roots of seedling plants. (**A**) Diagram to illustrate the root systems of cereal crops. Multiple crown roots (CRs) initiate first and second order lateral roots (LRs); single cell epidermal cells on the surfaces of these various root types can elongate to form root hairs (RHs). Scale bar represents 0.5 mm. (**B**) In textbooks and other literature, root hairs are frequently depicted as being present primarily in the “root hair zone”, a region proximal to the root tip.

In higher plants, the absorptive surface area of roots is increased with root hairs (RHs) which are subcellular outgrowths of epidermal cells [1]. When grown in soil, primary and adventitious roots, sometimes called crown roots (Figure 1A), have been reported to display a “root hair zone” proximal to the root tip (apex) where RHs initiate, elongate and mature (Figure 1B); RHs are reported to become fewer and/or less pronounced in older root segments closer to the soil surface [2,3]. Though the longevity of RHs has been poorly documented, in 1969, RHs of barley seedlings were shown to have a longevity of less than 5 days when grown in artificial germination pouches (envelopes) [4], which reinforced the concept of a canonical root hair zone even in cereals. As a result, today the RH zone concept is frequently presented in textbook diagrams and dogmatically taught to botany students [5,6]. However, as early as 1963, researchers have argued that such descriptions are over-simplified and that considerable variation in the RH pattern exists below ground [3]. Indeed, when the model plant *Arabidopsis thaliana* is grown in sterile agar media in Petri dishes as it produces small seedlings, RHs can be observed along the entire length of the primary roots [1]. Observed RH zones in field-grown plants may thus be due to mechanical forces in soil (e.g. shearing) along with catabolic biological processes (e.g. soil microbes) causing older, longer RHs to break, rather than an endogenous developmental program. New strategies are needed, however, to comprehensively phenotype RHs in large cereal crops.

### The challenge of phenotyping root hairs along the entire root system in large, cereal crops

There is significant interest by crop researchers to measure and optimize RH traits in major cereal crops in order to promote yield stability in low nutrient soils [7-10]. To overcome the technical challenge of studying RHs along expansive roots growing in soil, researchers have grown cereal crop roots in sand and gel substrates [8] or else sub-sampled a portion of the root system using laborious field excavation [9,11]. Aeroponic systems, in which roots are misted with a nutrient solution, have also been designed to prevent RH degradation, but the resulting RHs are significantly smaller than those grown in field soil [12].

### Turface® clay is an effective growth substrate to phenotype root hairs along entire crown roots in cereal crops

Here we present an alternative growth method, the use of coarse Turface® clay, which reliably preserves RHs along the entire length of crown roots (Figure 1A) in diverse cereal crops including wheat (*Triticum aestivum* L.), maize (*Zea mays* L.) and finger millet (*Eleusine coracana* L. Gaertn), the latter being a low-input crop grown by subsistence African/Asian farmers. Though Turface® is a well known plant growth substrate, used for decades to understand nodulation in legumes [13], to the best of our knowledge its use as a substrate to understand persistent RH growth in cereals has not previously been reported formally.

**Figure 2:**
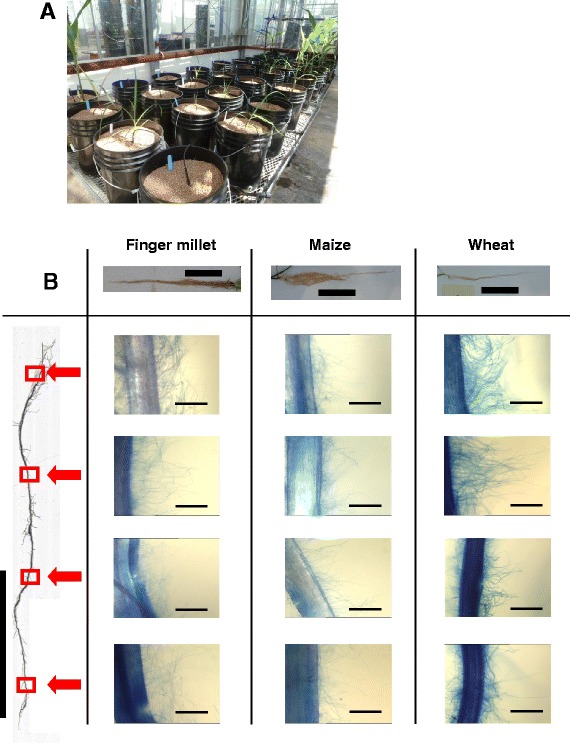
The Turface® fertigation growth system permits root hair growth along the entire crown root axis in cereal crops. (**A**) Picture of the greenhouse randomized complete block experimental design. (**B**) Root hair images from crown root segments of finger millet, maize, and wheat under 5x magnification. Microscopy scale bars represent 0.5 mm. All other scale bars represent 15 cm.

By using the Turface® fertigation growth system we were able to directly quantify RH length and density along the entire lengths of the crown roots of finger millet (Table 1), and we further verified that the technique could be applied to wheat and maize (Figure 2B). In all three cereals, RHs were visible along the entirety of all crown roots analyzed with the exception of the youngest, apical segment in some cases (Table 1, Figure 2B).

**Table 1:** **Length and density of root hairs along crown roots of finger millet**

			**Crown root segment**				
			**1**	**2**	**3**	**4**	**5**
**2012**	**Length (x100 μm)**	**Mean**	9.24	15.05	11.09	7.11	2.10
		**SEM**	0.47	0.28	0.37	0.24	0.10
		N*	200	199	200	160	143
	**Density (RHs/300 μm segment)**	**Mean**	14.55	15.65	16.15	11.25	6.20
		**SEM**	0.87	0.44	0.61	1.56	1.22
		N*	20	20	20	20	20
**2013**	**Length (x100 μm)**	**Mean**	9.91	11.42	9.17	4.18	2.84
		**SEM**	0.28	0.30	0.37	0.15	0.16
		**N**	200	200	198	139	91
	**Density (RHs/300 μm segment)**	**Mean**	14.15	14.55	13.45	7.20	2.65
		**SEM**	0.61	0.54	0.76	1.26	0.60
		N*	20	20	20	20	20

*N represents the number of microscope images from 5 plants used for analysis.

**Legend:** In 2012 and 2013, measurements were made of field-grown root hairs from the top of the crown roots (oldest, Segment 1) to the tips of the crown roots (youngest, Segment 5).

### On Turface®, root hairs continue to grow outside the root hair zone

Contrary to the RH zone concept, using Turface®, we observed RHs along the entire root network at several growth stages including full maturity – with the longest RHs being along the oldest crown root segments (Table 1, Figure 2B). RH density was also highest in this area (Table 1), though this may be due to selection bias imposed during visual ImageJ analysis. Our findings indicate that RHs in cereal crops can continue to grow well beyond what is typically thought of as the RH zone. Coarse Turface® clay thus stimulates and/or permits persistent RH growth.

### Possible reasons why Turface® clay permits or stimulates persistent root hair growth

Various authors have reported that the proliferation of RHs is influenced by the properties of the surrounding soil substrate. A RH infiltrates a macropore of soil [14], extending until the boundary of a soil cavity is reached at which point it adheres to the soil and performs roles in absorption and anchorage [15]. In coarse Turface®, particles range in size from ~0.5-3.3 mm providing an abundance of macropores. Soil moisture levels also heavily affect RH growth: dryness can desiccate RH cells while waterlogged soils are known to decrease RH length and density [16,17]. In our fertigation approach, the nutrient solution was administered with an automated irrigation system along with adequate drainage provided by Turface® itself; this system appears to have created an ideal micro-environment to encourage persistent RH growth. Lastly, Turface® is comparatively easy to remove from roots, meaning that fewer RHs are torn from the crown roots at harvest.

### Future perspectives

We have shown the research potential of the Turface® fertigation system by undertaking a highly robust characterization of finger millet’s RH profile along the crown root (e.g. 1730 individual measurements for RH length, Table 1). However, equally robust quantitative measurements should be undertaken to ensure that the growth system is similarly applicable to other crops, including wheat and corn. We do note that the root systems of all three crops qualitatively showed similar results, in terms of extensive RHs along all crown root segments, including outside of the canonical RH zone (Figure 2). The protocol we have outlined represents an intermediate between easily-studied agar-based growth systems, and agriculturally applicable field conditions. The Turface®-based methodology has the ability to preserve RHs even when cereal crops are grown to maturity which cannot be achieved in agar for such crops. While there are other systems such as aero- and hydroponics in which cereals can be grown to a late stage, published data shows conditions may be suboptimal for RH elongation in maize [12,18].

Despite the apparent promise of this methodology, it is an artificial system and there may be valid concerns about artifacts compared to growing cereal roots on soil. There may be two concerns in particular, the intrinsic characteristics of the RHs observed (e.g. length) and their presence outside of the canonical RH zone. With respect to the first concern, the observed range of average finger millet RH lengths (210 μm to 1505 μm, Table 1) when the plants were grown on Turface® is similar to those reported for barley cultivated on soil (~400 μm to 800 μm) and maize on sand (~500 μm to 1500 μm) [19,20]. These congruencies provide confidence that our system stimulates RH growth similar to more natural substrates.

With respect to the observed RHs outside the expected RH zone, we suggest that the RH zone concept should be re-examined critically under field conditions. While the growth system used here was artificial, there may be natural conditions (e.g. gravelly, well-drained soils in regions with high precipitation) in which plants might similarly display the ubiquitous root hair phenotype observed in this study. It will be especially interesting to understand the physiology of older RHs compared to those in the canonical RH zone, for example by analyzing markers for normal RH function. However, at present this cannot be accomplished in finger millet due to the lack of available sequence data and functional experiments.

The literature describing RH processes in crop plants is scarce, with the majority of RH physiology and genetics research having been conducted in *Arabidopsis*, including the role of calcium gradients and cytoskeleton dynamics [21,22], the existence of a basipetal auxin gradient associated with RH initiation [23], and the role of AUX1 and PIN family influx and efflux auxin pumps [23-26]. The ability to study the impact of diverse alleles of orthologous genes on RHs in mature cereal crops has the potential to open novel avenues for crop improvement.

### Field growth system methodology

Experiments were conducted over two growing seasons in 2012 and 2013 at the Arkell Research Station (43°53′N, 80°18′W, 325 m above sea level) near Guelph, Canada. Finger millet seeds were germinated in a laboratory at room temperature prior to transplantation at the field site. Experimental units consisted of single finger millet plants grown in 22 L plastic pails (28 cm in diameter) filled with an inert, coarse Turface® MVP clay (Profile Products LLC., Buffalo Grove, Ill), as per a field fertigation system described previously [27]. Millet plants were irrigated zero to three times per day, as required. A modified Hoagland’s solution [27] (minus nitrogen) was stored in a 340 L lidded plastic pail and diluted with water at a ratio of 1:100 at the time of application. The pH of the diluted nutrient solution was adjusted to a range of 6.5-6.7 with the addition of HCl. Each pail was fitted with two fertigation tubes, known to deliver a minimum of 100 ml of nutrient solution over a 10 min interval [28]. As the original point of the experiment was to examine the effects of nitrogen limitation, nitrogen was provided separately by hand as 13 weekly doses of 5 kg total N/ha as urea granules dissolved in 1 L H_2_0.

At plant maturity in both years, five root systems were stored at −20° C in 50% ethanol until analysis, at which point they were thawed and subjected to microscopy. The longest representative crown roots were selected from each plant. Five segments measuring 1 cm in length were cut from each crown root at equal distances along the root and rinsed in ddH_2_0. Segments were stained with 0.4% Trypan Blue solution (MP Biomedicals LLC, Solon, OH) as reported in other studies [9] for 10 minutes, rinsed and examined with a Leica MZ8 stereomicroscope (Leica Microsystems GmbH, Wetzlar, Germany) under 5× magnification. Northern Eclipse software (version 5.0, Empix Imaging Inc., Mississauga, Canada) was used to capture four images of each root segment with a Sony DXC-950P Power HAD 3CCD color video camera (Tokyo, Japan). Using ImageJ (Version 1.47, Wayne Rasband, NIH, USA), RHs were quantified for length and density by first calibrating the program to a scale image 1 mm long. Ten RHs from each image were traced to obtain representative RH length data for that segment, while the number of RHs within a 300 μm segment were counted to obtain representative RH density data (Table 1). Hence, the RH length data was derived from attempts to measure 10 RHs from each of four images per crown root segment, with 5 biological replicates (therefore maximum n = 200 root hairs per root segment). Column statistics were generated using GraphPad Prism® software (version 6.04; GraphPad Software, Inc., CA, USA).

### Greenhouse growth system methodology

To verify that the Turface® system could be applied to phenotype root hairs in diverse cereals, spring wheat (cv. Quantum, C&M Seeds, Palmerston, Canada) and a maize hybrid (CG60 × CG102) were grown in Turface® in a greenhouse using a randomized complete block design, with a commercial variety of finger millet as a positive control (Figure 2). All other fertigation conditions were identical to those noted above. Crown roots of all three species were examined with microscopy exactly as described above with the exception that four segments were examined per plant instead of five.

## Abbreviations

RHs: Root hairs
CR: Crown root
LR: Lateral root
